# How has the COVID-19 pandemic affected young people?—Mapping knowledge structure and research framework by scientometric analysis

**DOI:** 10.3389/fpubh.2022.1052727

**Published:** 2022-12-02

**Authors:** Xiangfei Li, Jiahui Yu

**Affiliations:** School of Economics and Management, Tiangong University, Tianjin, China

**Keywords:** COVID-19, teenager, adolescent, children, mental health, lifestyle, VOS viewer, CiteSpace

## Abstract

Since the outbreak of COVID-19, there has been a large body of literature focusing on the relationship between the COVID-19 pandemic and young people. The purpose of this study is to explore the current research status and the specific mechanism of COVID-19's effects on young people based on related literature. This paper mainly used VOS viewer and CiteSpace software to conduct a scientometric analysis of 5,077 publications retrieved from the Web of Science database. The results show that the main contributors to the field were mainly from North America and Europe, and the trend of research focus was from shallow to deep. The five main research areas in the field were summarized by keyword clustering analysis as follows: lifestyle changes due to lockdown; changes in stress and emotions; psychological illness and trauma; risk perception and practice toward the epidemic; interventions and social support. Finally, they were linked by four pathways to form a framework that integrates the relationships between the five domains and between elements within each of them, revealing the mechanism of COVID-19's effect on young people. In addition, less studied but promising elements are also presented in the framework, such as research on special groups (disadvantaged socioeconomic groups and sexual minority youth) and extreme suicidal tendencies that deserve our further attention.

## Introduction

Since the emergence of the Corona Virus Disease 2019 (COVID-19), the virus has rapidly swept the world due to its high infectiousness. As of September 2022, there were more than 600 million cumulative confirmed cases and more than 6 million deaths worldwide (1). Countries around the world have responded with various measures to control the spread of the virus. And in many cases, people have to reduce their social activities and keep a social distance. For this group of young people, social isolation (such as the closure of schools and social venues) has kept them in a state of long-term online learning and weakened their connections with teachers, classmates, and other social networks. At the same time, emerging adults who are about to enter society and seek employment also feel confused and uneasy about their futures (2). With COVID-19 now in its third year, the prolongation of such a public crisis event generates fear, sleep disorders, anxiety, depression, and other risk factors that affect the mental health of young people (3–5) and can even lead to unhealthy lifestyles, reduced physical fitness, and other physical problems in the future as a result (6).

So far, a large amount of literature has been published in this field to investigate and analyze the effects of COVID-19 on young people, and it can be said that the relationship between the COVID-19 pandemic and young people deserves our attention. However, the scope of research involves different countries and regions, the content of research is abundant, and the focus of research is different. In the face of the vast literature, some meta-analyses or reviews have existed, but most of them are relatively one-sided, and there is a lack of more comprehensive and systematic studies. Scientometrics, as a quantitative research method, enables a systematic and objective analysis of a large number of publications to identify the current development status and research focus in a particular field (7, 8). In this paper, we will collect and organize the literature in this field in the Web of Science database through the corresponding search strategy and visualize the retrieved literature by using VOS viewer and CiteSpace. The goal of this paper is to provide this field with a new way to understand knowledge distribution through visual analysis and to summarize the research status, hotspots, and main research framework in this field.

Compared with previous review articles on this topic, there are three main contributions as follows: (1) The current state of research (number of publications and leading countries, institutions, subject categories, journals, and references), research focus, and research trends are visualized to provide a new approach to understanding the field from a scientometric perspective. (2) Through keyword clustering analysis, five main research domains within the field are identified, and based on this, they are linked by four pathways to form a framework showing the influence relationships between them and their respective internal elements, thus revealing how the COVID-19 pandemic has affected young people. (3) Studies that are difficult to be noticed due to less research but still have the prospect of development (e.g., suicidal extremism, special groups such as disadvantaged socioeconomic groups and sexual minority youth) are also captured and presented in the framework.

## Materials and methods

### Data sources and search strategies

The Web of Science Core Collection (WoSCC) was selected as the data source for this study, with a large number of publications covering the most comprehensive range of literature possible. The search date for this study was November 4, 2022, and the search date range was 2020-01-01 to 2022-09-30, with the search term “TS = (COVID-19 OR SARS-CoV-2 OR Coronavirus disease 2019 OR severe acute respiratory syndrome coronavirus 2) AND TS = (teenager OR adolescent OR juvenile OR young OR child OR children OR offspring OR descendant OR junior OR son OR daughter).” After the search, the document types were restricted to “review” and “article,” and the language was restricted to English. In order to ensure that the results are more accurate and effective, after discussion with experts from related disciplines, it was determined that the Web of Science Categories should be limited to Public Environmental Occupational Health, Health Care Sciences Services, Psychology Multidisciplinary, Psychology Developmental, Psychology Clinical, Education Educational Research, Health Policy Services, Social Sciences Interdisciplinary, Social Work, Psychology Social, Sport Sciences, and Psychology Social. Then, the two authors continued to conduct manual screening to eliminate publications that were not related to the research theme. Finally, after deleting duplicate records, a total of 5,077 records were obtained, including 4,851 original research articles and 226 reviews. In summary, authoritative database sources, comprehensive search terms, rigorous category screening, and ultimately manual screening can effectively ensure the accuracy of the study sample.

### Scientometric tools and functions

#### Visualization software

VOS viewer is a free JAVA-based software that focuses on the visualization of scientific knowledge through the use of literature data (9), and the version used in this article is VOS viewer1.6.17. We used the citation, co-citation, and co-occurrence functional modules of the software to visualize and analyze institutions, journals, references, and keywords. Each node in the visualization map represents a different parameter, such as institution, journal, keyword, etc. The larger the node, the greater its weight. Nodes are connected by links. Total Link Strength (TLS) is an important metric for identifying this type of visual graph, and the TLS value of a node refers to the number of times that node appears together with all other nodes (10). CiteSpace is a JAVA application for presenting the structure, patterns, and distribution of scientific knowledge (11). The version used in this article is CiteSpace V6.1.R2. The program focuses on analyzing the research progress in a field by using information such as literature authors, titles, keywords, abstracts, citations, etc. In this study, we used CiteSpace to mine the data and produce the dual-map overlay of the journals and the network visualization map of subject categories.

#### Visualization process

In the process of visual analysis by VOS viewer software, the basic parameters were set as follows: The visualization scale was set at 1.0, and the weight of the node size was total link strength; the layout used the default value; the algorithm for the clustering process using this software was the association strength of the normalization method, as well as a clustering resolution of 1.00 and a minimum cluster size of 1. Based on the above parameter settings, we performed citation analysis of institutions, co-citation analysis of journals and references, and co-occurrence analysis of keywords. The number of clusters in each visualization map will be automatically generated by the software's association strength algorithm.

This article used CiteSpace software to make the dual-map overlay of the journals, set the source circle size to 120, set the target circle size to 15, and checked Z scores. In the process of creating the visualization map of subject categories, the following parameters were set: Time Slicing was 2020 to 2022; Node Type was chosen as categories; Pruning was checked as pathfinder and pruning sliced networks; the rest were the default settings.

## Results and discussion

### Knowledge structure of research status

#### Publications and geographic distribution

**Figure 1:**
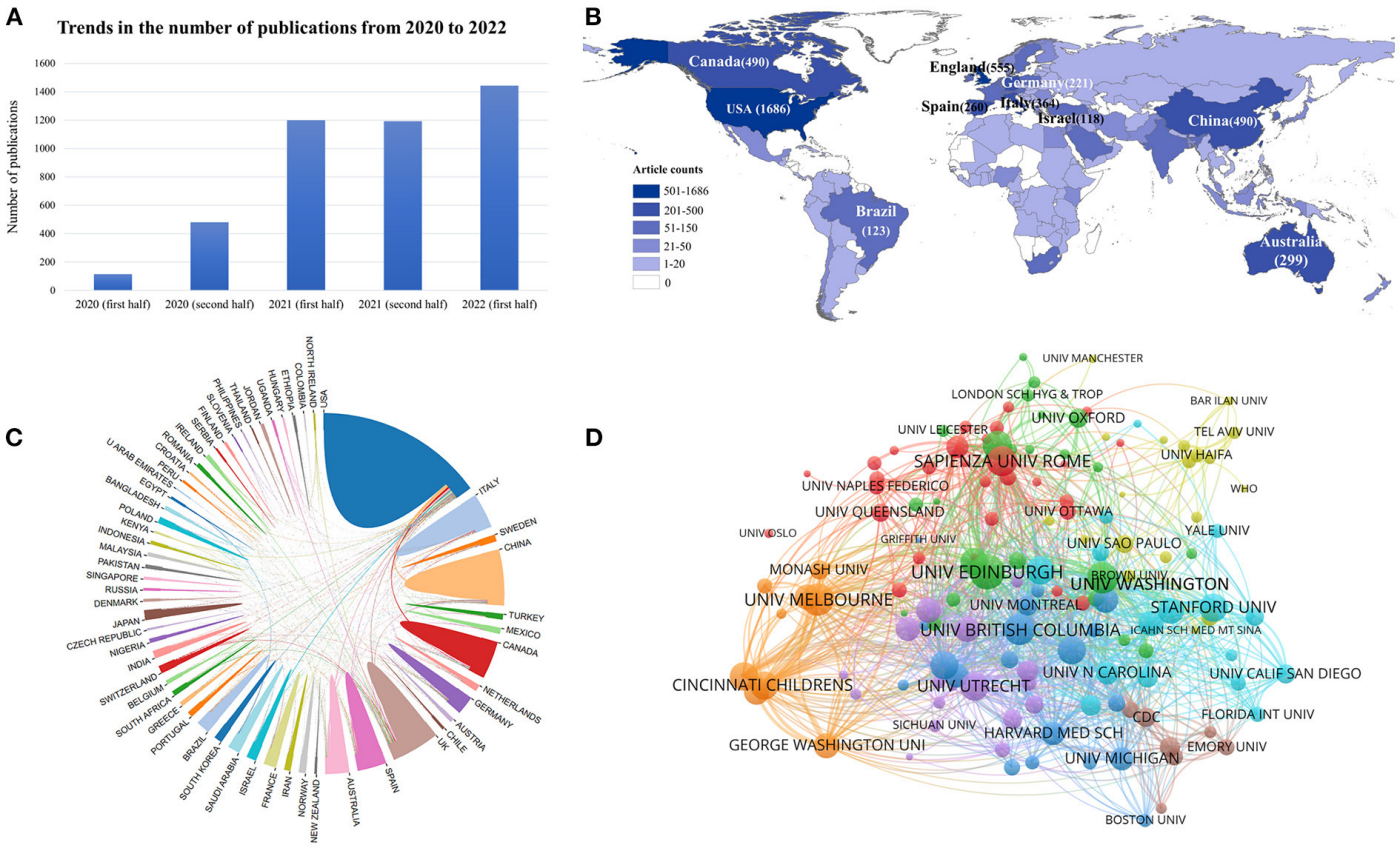
**(A)** Trends in the number of publications from 2020 to 2022. **(B)** Geographical distribution based on the number of articles issued by various countries/regions. **(C)** Inter-country/region cooperation relationship map. **(D)** Citation network visualization map of institutions.

Figure 1A presents the trends in the number of publications from 2020 to 2022 on a half-yearly basis. Overall, there is a growing body of research exploring the relationship between COVID-19 and young people: from 2020 onwards, the number of publications starts to increase significantly; from the second half of 2021 to the present, the upward trend plateaus. A small increase in the number of publications in this area is expected in the second half of 2022. From the search results, a total of 156 countries/regions have published articles in this research area. The country/region with the highest number of publications and citations was the USA, and its number far exceeded that of other countries, followed by England and China.

Figure 1B shows a geographical distribution based on the number of articles issued by various countries or regions in the field of research on the relationship between COVID-19 and young people. This map allows us to visualize that the majority of articles came from North America, Western Europe, and East Asia. The inter-country/region cooperation relationship map (Figure 1C) shows the international partnerships between countries/regions with at least 20 publications by connecting the lines of different colors. We can clearly see that the USA and the UK were the countries that cooperated the most with other countries.

A total of 6,226 institutions have published articles related to this topic. Table 1 shows the top 10 most active institutions. Overall, these prolific institutions were mainly from Europe and North America. Figure 1D shows the citation network visualization map of institutions. By limiting the documents of the institutions to at least 15, this figure presents a total of 140 nodes representing institutions and 3,290 links. The institution with the highest TSL value was the University of Edinburgh from England (TLS = 324), which had a strong influence among the institutions. Followed by the University of Melbourne (TLS = 288) from Australia and the University of Washington (TLS = 270) from the USA.

**Table 1:** The top 10 most active institutions.

**Rank**	**Institutions**	**Continents**	**Article counts**	**Total citations**	**Average citations per article**
1	University of London	Europe	191	3,408	17.84
2	University of California System	North America	182	1,406	7.73
3	Harvard University	North America	112	1,327	11.85
4	University College London	Europe	104	2,701	25.97
5	University of Toronto	North America	93	701	7.54
6	Pennsylvania Commonwealth System of Higher Education (PCSHE)	North America	75	593	7.91
7	State University System of Florida	North America	71	1,276	17.97
8	University of North Carolina	North America	67	580	8.66
9	University of Melbourne	Oceania	65	447	6.88
10	University of Texas System	North America	63	402	6.38

#### Analysis of subject categories and interdisciplinary

**Figure 2:**
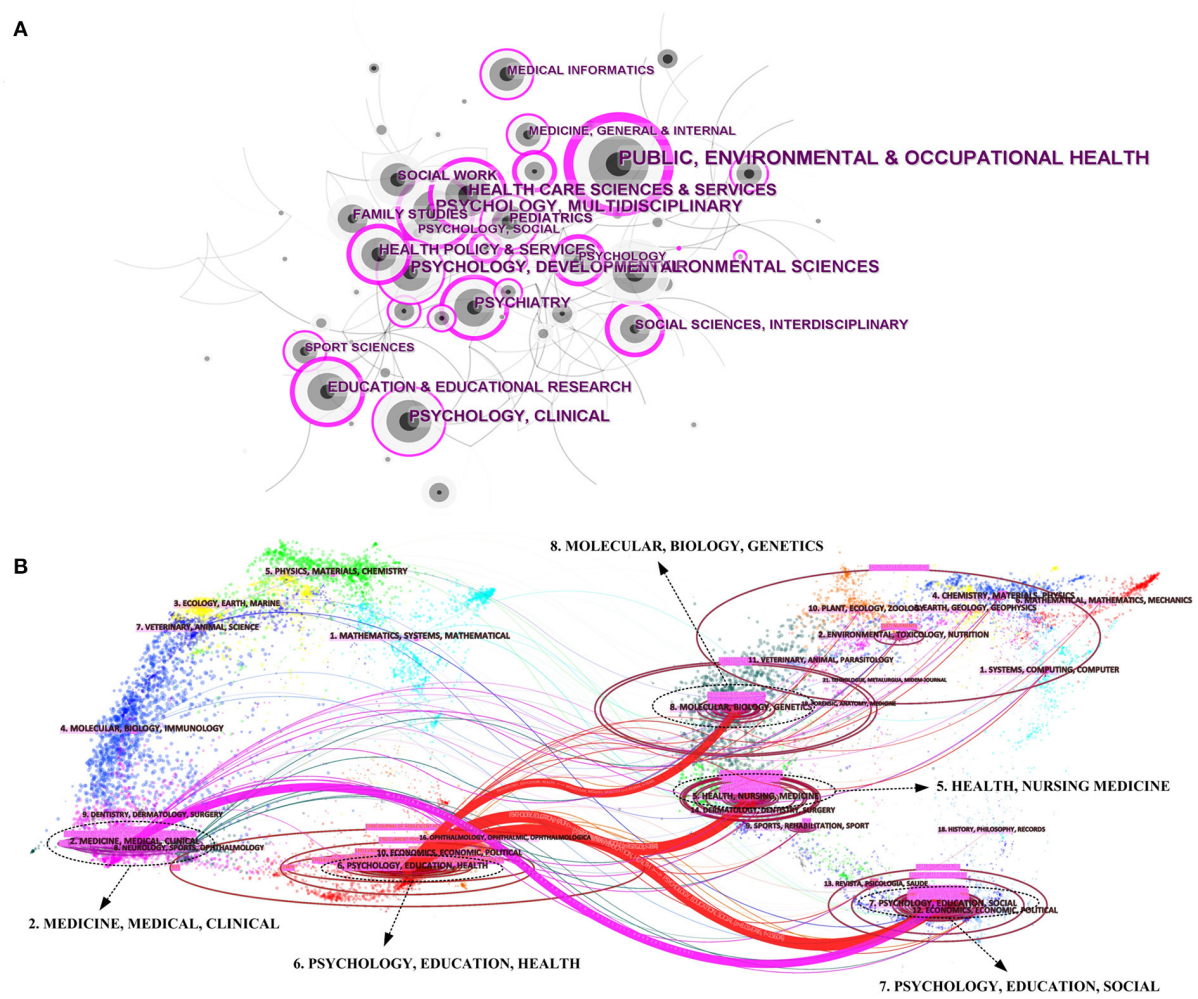
**(A)** The network visualization map of subject categories created with CiteSpace software. **(B)** The dual-map overlay of the journals.

In this paper, we analyzed the subject categories obtained from the search results using CiteSpace software and created a network visualization map consisting of several nodes representing the subject categories shown in Figure 2A. Nodes with a centrality value >0.1 are key nodes and carry a purple outer ring. The higher the centrality value of a node, the greater its influence. The top three disciplinary categories with the highest centrality values were PUBLIC, ENVIRONMENTAL and OCCUPATIONAL HEALTH (0.7), PSYCHOLOGY, MULTIDISCIPLINARY (0.29), and PSYCHIATRY (0.28).

The dual-map overlay of the journals (Figure 2B) reflects the topic distribution of journals, which are clustered and named by the LLR algorithm. In the final result presentation, the citing graph of journals is on the left, the cited graph of journals is on the right, and the curves are the citation links (12, 13). On the left side, the focus was on the topics “6. PSYCHOLOGY, EDUCATION, HEALTH” and “2. MEDICINE, MEDICAL, CLINICAL,” which were connected to “5. HEALTH, NURSING, MEDICINE,” “7. PSYCHOLOGY, EDUCATION, SOCIAL,” and “8. MOLECULAR, BIOLOGY, GENETICS” on the right side by four connecting lines. These four lines were the most dominant citation paths among journals in this field. And on the right side of the graph, it can be seen that the topic distribution of cited journals was more dispersed.

#### Authoritative journals and highly cited references

**Figure 3:**
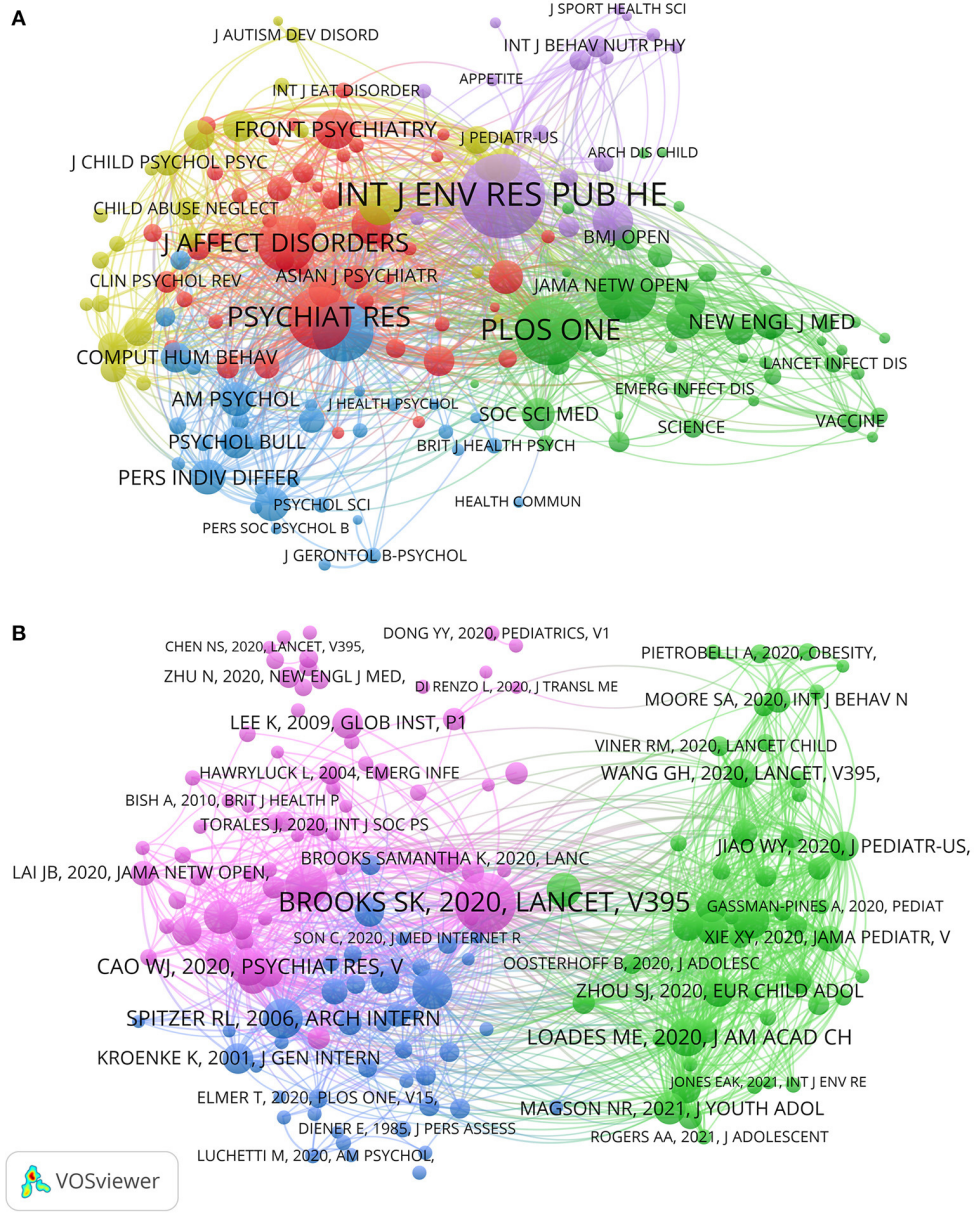
**(A)** Co-citation network visualization map of journals by VOS viewer. **(B)** Co-citation network visualization map of references by VOS viewer.

The journals with the most contributions are listed in Table 2. Figure 3A shows a visualization of the co-citation network of journals, which presents the journals with at least 180 citations in the field, forming a total of 170 nodes with 14,188 links in the graph. The top three journals with the largest TSL values were *INTERNATIONAL JOURNAL OF ENVIRONMENTAL RESEARCH AND PUBLIC HEALTH* (TSL = 96,741), *PLOS ONE* (TSL = 68,082), and *PSYCHIATRY RESEARCH* (TSL = 61,294). These three journals had the strongest correlation with other journals in the field.

**Table 2:** The journals with the most contributions.

**Rank**	**Journal title**	**Publications**	**IF (2021)**	**JCR (2021)**	**Total citations**
1	International Journal of Environmental Research and Public Health	685	4.614	Q1/Q2	6,868
2	Frontiers in Psychology	292	4.232	Q1	3,233
3	Frontiers in Public Health	208	6.461	Q1	1,224
4	BMC Public Health	130	4.135	Q2	1,328
5	Journal of Adolescent Health	104	7.83	Q1	2,870
6	Healthcare	77	3.16	Q2	250
7	Current Psychology	70	2.387	Q3	309
8	Journal of Medical Internet Research	54	7.076	Q1	1,162
9	European Child Adolescent Psychiatry	40	5.349	Q1/Q2	1,415
10	Social Science Medicine	33	5.887	Q1/Q2	590

Figure 3B shows the co-citation network visualization map of references. One hundred fifty-eight references with at least 40 citations were filtered out to form this visualization map. Among them, the reference with the largest TLS value was *The psychological impact of quarantine and how to reduce it: rapid review of the evidence* (14) published in 2020 by Brooks SK et al. in the *Lancet*, which shows that this article had the most associations with other articles.

### Research focus and trends

#### Changes in co-occurrence keywords

**Figure 4:**
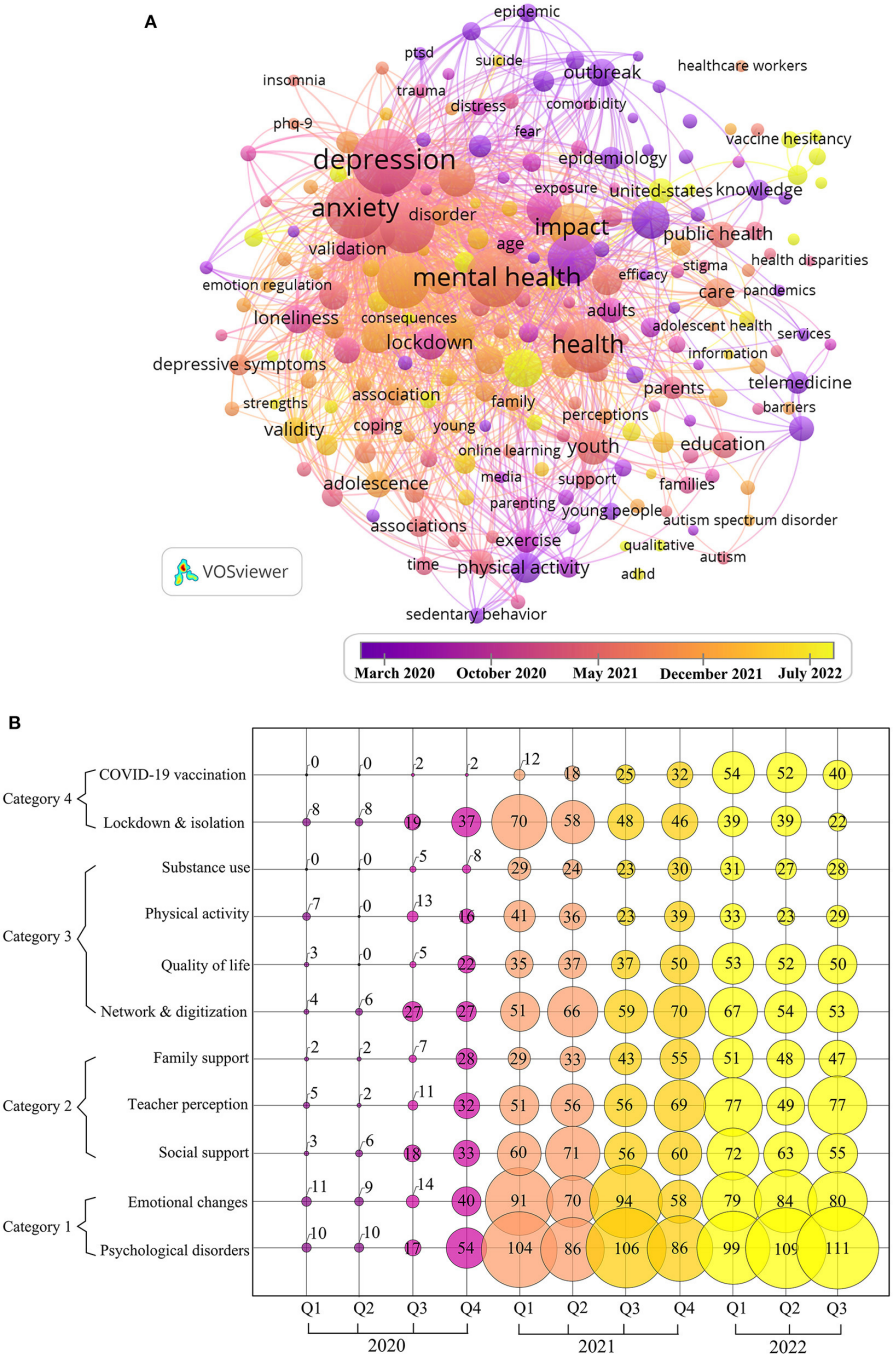
**(A)** Co-occurrence overlay visualization map of high-frequency keywords. **(B)** Bubble charts of research topics.

The analysis of high frequency co-occurrence keywords not only identifies the research focus but also reveals the changing themes and trends. We filtered keywords that occurred 30 or more times as high-frequency keywords and excluded keywords that were duplicated with the search terms to finally generate the co-occurrence overlay visualization map (Figure 4A).

In Figure 4A, the keywords with the largest nodes, i.e., the most co-occurrences, are “depression,” “anxiety,” “mental health,” “stress,” “impact,” “prevalence,” etc., indicating that most of the research in this area has focused on mental health. The color of each node in the figure is determined by the average appearing year (AAY), and the color of the nodes with earlier appearing years is purple, while the color of the nodes with later appearing years is yellow. The number of keywords obtained for 2020, 2021, and the first half of 2022 were 2,140, 6,580, and 4,481, respectively, indicating that the scope study is expanding rapidly and showing a further growth trend.

At the beginning of the epidemic in 2020, research was focused on: “outbreak,” “social isolation,” “quarantine,” “outcomes,” “fear,” “worry,” “mortality,” etc., which reflects the harm caused by the spread of the epidemic itself and the intuitive feelings it brought to young people. In 2021, the co-occurrence keywords were mainly focused on words like “mental health,” “anxiety,” “depression,” “disorders,” “prevalence,” and “symptoms,” etc. This indicates that medium term concerns were more serious psychological affects. Since entering 2022, keywords such as “therapy,” “vaccine,” “sex,” “ADHD,” “suicide,” “predictors,” and “adjustment” have emerged. People's attention has shifted from the initial outbreak itself to the treatment of the harm caused by the outbreak and more in-depth and detailed research, such as the investigation of the hesitancy of young people regarding vaccines (15, 16), the study of the sexuality of adolescents and young adults (17, 18), and the study of the impact of the epidemic on children and adolescents with ADHD (19).

Cumulatively, studies of the impact of COVID-19 on young people show a trend from superficial to deeper, evolving from focusing on the initial stage of the outbreak to exploring more subtle changes.

#### Evolution of research topics

After sorting the keywords by frequency and focusing on the top 100 most frequently occurring keywords, a bubble chart of this study area by quarterly changes between January 2020 and September 2022 is shown in Figure 4B with 11 topics and 4 main categories, which can visually display the change in the heat of the main research hotspots in the field through the evolution of time.

The content of Category 1 “mental health” (“emotional changes” and “psychological disorders”) is the most researched, indicating that the mental health of young people has been receiving great academic attention from 2020 to 2022, and its content mainly focuses on issues such as depression, anxiety, stress, and loneliness in young people. Although interest declined at the end of 2021, it rebounded rapidly afterward.

Category 2 focuses on “support from various groups.” Except for the topic “teacher perceptions,” the popularity of other topics in this category has decreased in 2022.

Category 3 mainly discusses “impact on lifestyle.” The main components of the topics “physical activity” and “network & digitization” are the investigation and confirmation of the decrease in physical activity and the increase in internet use among young people during the epidemic (20). The year 2021 is the culmination phase of these topics, followed by a decline, suggesting that academic interest in these topics is declining. However, the topics “substance use” and “quality of life” in category 3 have not fluctuated much in the past 2 years and will continue to receive attention in the future.

Category 4, “epidemic control,” includes topics such as “lockdown and isolation” and “COVID-19 vaccination.” “Lockdown and isolation,” which focuses on the outbreak itself and the immediate impact of lockdown and isolation on young people, has received considerable attention but is now less and less studied. Meanwhile, the popularity of the COVID-19 vaccination has increased in 2022. This is because, since the emergence of the COVID-19 vaccine as one of the major measures for people to deal with COVID-19, its safety and efficacy, as well as young people's attitudes toward the vaccine, have been the subject of research.

### Main research domain and framework

#### Cluster analysis of keywords

**Figure 5:**
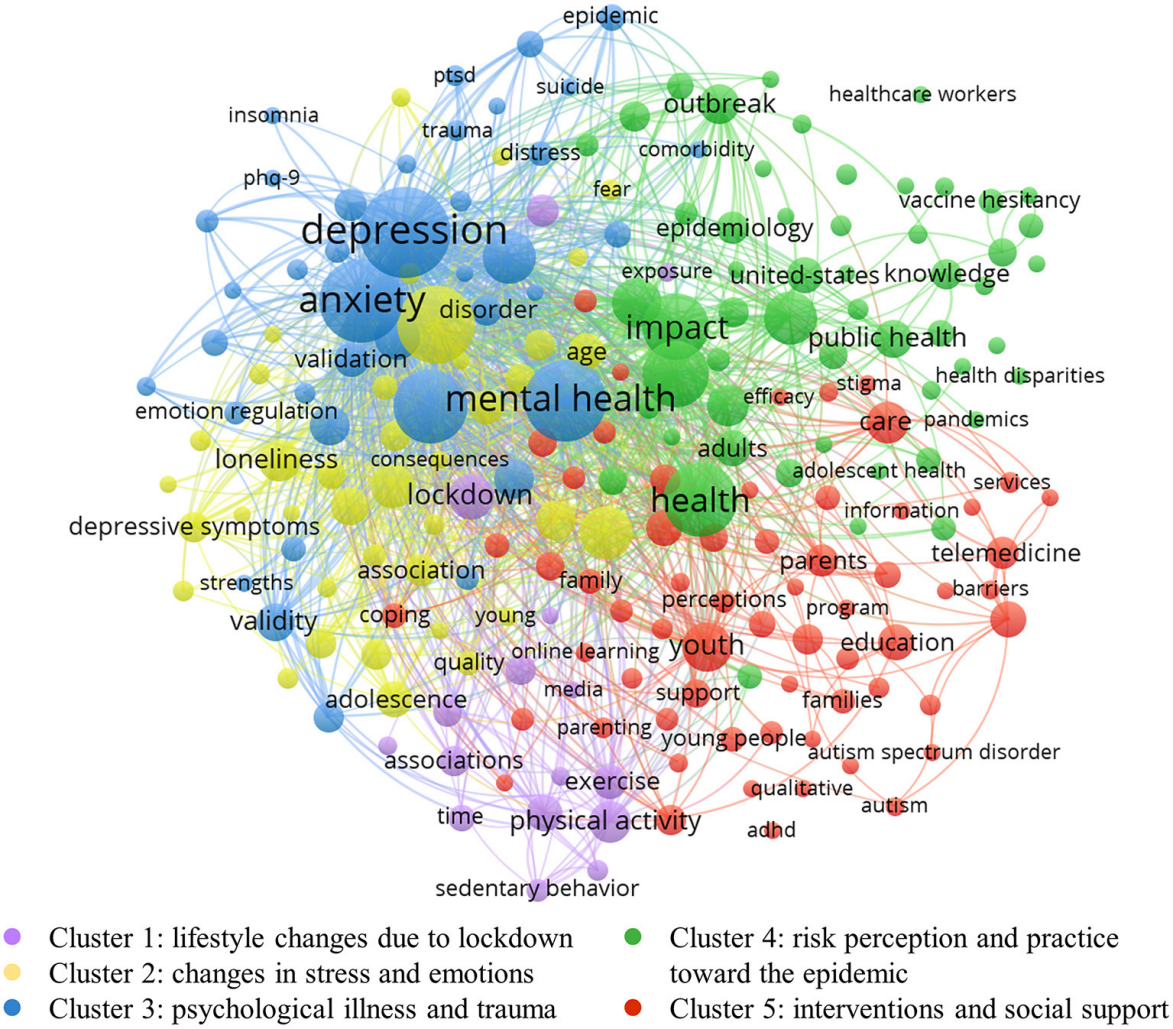
Keyword network map consisting of five clusters.

Cluster analysis of frequently occurring keywords facilitates a precise grasp of the main knowledge distribution in the field, which is essential for understanding hotspots and anticipating trends. In this study, the keywords were visualized and clustered by VOS viewer. The number of keyword occurrences was limited to 30 or more, and duplicate or unreasonable keywords were manually removed, thus forming the keyword network visualization map (Figure 5). Based on the association strength algorithm, the software divided the high-frequency keywords into five clusters. However, over-reliance on the content of high-frequency keywords for the analysis may result in the five clusters obtained from the results being difficult to achieve comprehensive coverage of all information or having partial overlap between the clusters. Therefore, after understanding and analyzing the five clusters, we imported the data into CiteSpace for keyword clustering analysis. Then, after verifying the Silhouette score several times, we confirmed that the five clusters here are all reasonable. Finally, by inviting three experts in adolescent mental health and two experts in youth sports to study and discuss the keywords in each cluster, we named each of these five clusters, which are shown below:

**Cluster 1** (lifestyle changes due to lockdown, purple nodes): The main keywords included in this cluster are: “lockdown,” “physical activity,” “screen time,” “substance use,” “wellbeing,” etc. Lockdown is one of the essential preventive and control measures during the COVID-19 pandemic to prevent the transmission and spread of the epidemic. However, because of the social isolation and school closures, the living conditions of young people are very different compared to those of the past. From several of the most significant nodes in the cluster, it can be summarized that the research in this cluster focused on the changes in physical activity, internet use, and substance use among young people as a result of the lockdown under COVID-19.

**Cluster 2** (changes in stress and emotions, yellow nodes): The prominent keywords in this cluster are: “stress,” “loneliness,” “emotion,” “fear,” etc. Children and adolescents are still immature in their physical and psychological development and have a limited ability to cope with external stress and stabilize their emotions, while young people entering society are also facing a volatile situation pattern. Therefore, the changes in stress and emotions among young people during the epidemic have received much attention and have led to a series of related studies, which is another major research area in this field.

**Cluster 3** (psychological illness and trauma, blue nodes): This cluster reflects the increased stress and emotional distress associated with the COVID-19 pandemic, leading to the onset of some psychological illness and trauma in young people. The majority of the literature in this area highlights the impact of COVID-19 as a health crisis on the development of psychological illness in young people, which is one of the most important and typical topics in these clusters. The main keywords included in this cluster are: “mental health,” “depression,” “anxiety,” “disorders,” “PTSD,” etc.

**Cluster 4** (risk perception and practice toward the epidemic, green nodes): the main keywords included in this cluster are: “risk,” “health,” “attitude,” “knowledge,” “vaccination,” etc. The high risk of COVID-19 puts people at constant risk of infection, but subjective risk perceptions of the epidemic often vary from person to person. Young people's risk perceptions of outbreaks influence their outbreak prevention behaviors and mental health status, and this area has received attention in academic circles.

**Cluster 5** (interventions and social support, red nodes): COVID-19 and its attendant controls have a significant impact on young people, so another major area of research on how to provide appropriate early interventions and strong social support for young people is presented in this cluster. The main keywords included in this cluster are: “intervention,” “support,” “service,” “education,” “parents,” etc.

#### The mechanism of COVID-19's effect on young people

**Figure 6:**
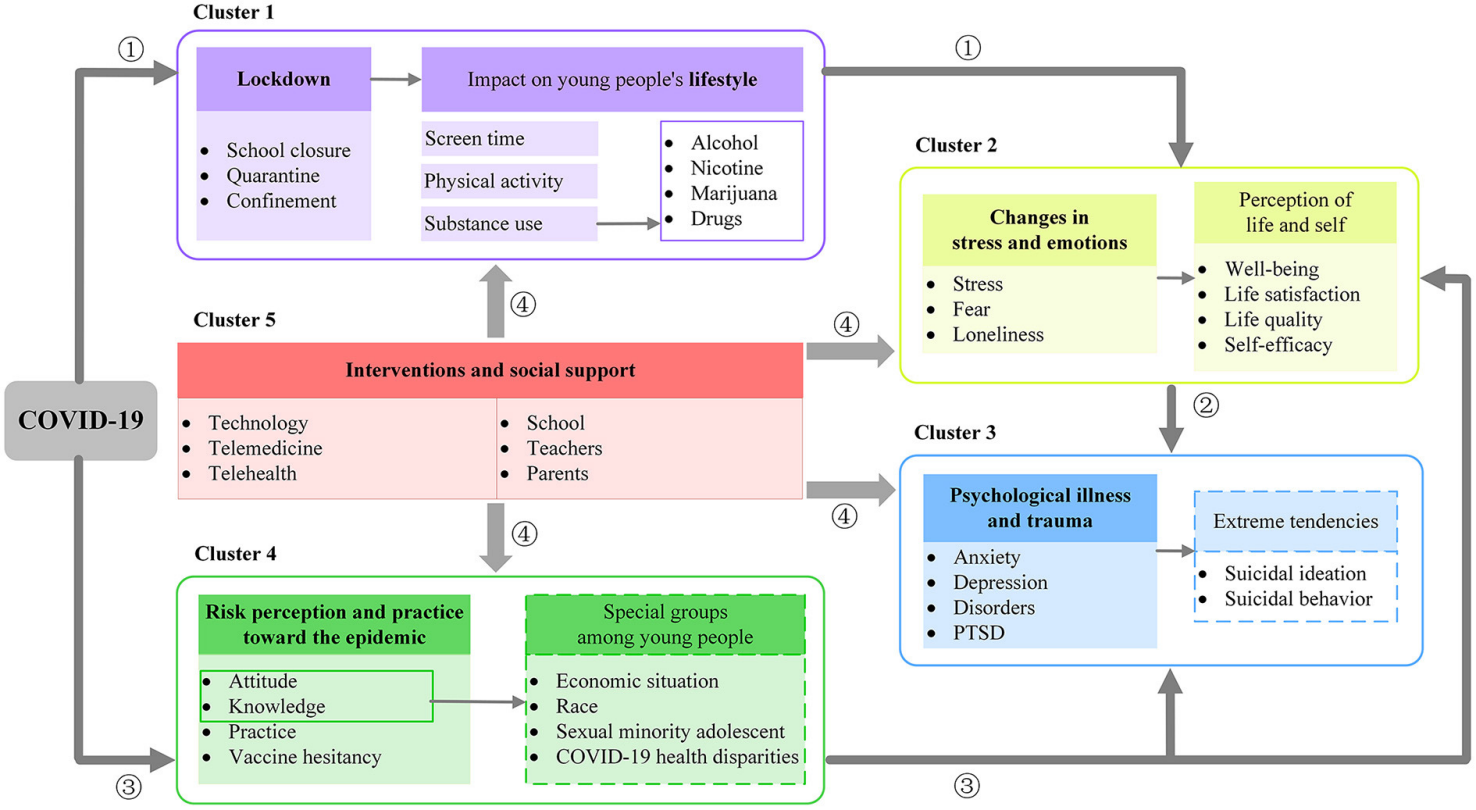
The framework of the effect of COVID-19 on young people, based on high-frequency keywords and cluster analysis.

By reading and sorting out the representative literature involved in each cluster, we identified the relationships among the clusters and produced the framework of the effect of COVID-19 on young people (Figure 6). The colored boxes in the figure coincide with the colors to which the clusters belong in Figure 5. The text in the dashed box indicates that it is not represented in the co-occurring keyword clusters due to the small number of existing studies, but it still deserves our further attention.

##### Mechanism between the five clusters

In Figure 6, there are four pathways expressing the relationship between these five clusters in the effect mechanism. Firstly, pathway 1 shows that the global outbreak of COVID-19 has caused countries and regions to adopt lockdown measures to deal with this highly infectious virus, which has changed the lifestyles of young people (**Cluster 1**). The disruption of the formerly standard and healthy lifestyle of young people has had a consequent impact on their mental health status. Secondly, pathway 2 is a shift from **Cluster 2** to **Cluster 3**. That is, increased stress and fluctuating emotions (e.g., the emergence of adverse feelings such as fear, loneliness, etc.) in young people during COVID-19 further intensify into serious psychological disorders and severe trauma (e.g., anxiety, depression, PTSD, etc.). Pathway 2 reflects this exacerbation process. In addition, from pathway 3, the perceived risk profile and action performance of young people in the context of the COVID-19 pandemic are also a major research component (**Cluster 4**). The level of risk perceived by young people facing such a major public health hazard and the uncertainty and uncontrollability of that risk will increase the psychological stress and negative emotions of individuals and have an impact on their mental health. Finally, pathway 4 flows to the other four clusters centered on intervention and social support for young people (**Cluster 5**). Given that the aforementioned COVID-19 has led to lifestyle changes, severe risk perceptions of the epidemic, increased stress, mood swings, and psychological disorders among young people, interventions and strong social support are needed to address the current unpromising situation of young people.

We focus on the specific mechanisms of effect within each cluster as follows.

##### Effect mechanisms within each cluster

###### Effect mechanism in cluster 1: Lifestyle changes due to lockdown

The impact of the lockdown measures on young people's lifestyles is reflected in the purple box in Figure 6. First, there has been a general increase in screen time use by young people who are confined to their homes due to the need for online learning and contact with family and friends *via* the Internet (20, 21). However, with the increase in screen time, game addiction and compulsive internet use have been exacerbated, especially among adolescents and young adults (22, 23). This may be a risk factor for worsening sleep quality, increased stress, and the development of depression and anxiety symptoms (24–26). Second, it is reflected in the reduction of physical activity among young people as a result of the lockdown, which has now been confirmed by numerous studies (27–29). More exercise can be effective in protecting one's mental health, such as preventing the emergence and development of anxiety and depression (30, 31), but the current situation of young people today is deviating from this. Third, in the context of COVID-19, substance use among young people has changed (32, 33). Since the beginning of social distance, studies have found increased alcohol and marijuana use among adolescents (34, 35), and there are also studies linking the emergence of the COVID-19 pandemic to increased nicotine use and prescription drug abuse (36). For young people in the early exploratory stages of substance use, uncontrolled substance use during COVID-19 has the potential to lead to the emergence of substance use disorders, increased dependence, and poorer mental health (37, 38).

###### Effect mechanism in cluster 2: Changes in stress and emotions

Children and adolescents are one of the most vulnerable groups in this pandemic in terms of the development of psychological abnormalities (39, 40). It was found that their stress during this epidemic mainly stemmed from the inability to participate in social activities/normal daily activities and important plans/events being canceled or postponed (41). Children and adolescents who did not originally have mental health disorders experienced a significant deterioration in mood during the pandemic (42), and feelings of anxiety and sadness were prevalent among them (43). It can be argued that the increased stress and unstable emotions caused by COVID-19 may reduce young people's quality of life and life satisfaction, affecting their daily wellbeing. Also, young adults exhibit strong concerns and feelings of loneliness during the COVID-19 pandemic (44, 45), which would predict the emergence of certain mental health symptoms. Compared with older adults, young adults are more susceptible to stress-induced mood swings and respond to stressful situations with fewer resources and experience. Their greatest stress stemmed mainly from uncertainty, such as not knowing when the COVID-19 pandemic will end (41). Young adults who have just reached adulthood are likely to be confused and worried about their future in the face of a difficult college life and a worsening economic environment, which could have a serious impact on their self-efficacy and undoubtedly reduce their sense of wellbeing.

###### Effect mechanism in cluster 3: Psychological illness and trauma

Overall, the prevalence of anxiety and depression was much higher than before the COVID-19 pandemic (41, 46, 47), and more pronounced in females (41). The COVID-19 pandemic is sufficient as a life-threatening infection to cause post-traumatic stress disorder (PTSD), which may have more severe consequences for children and adolescents (48, 49). Young people's unattended and unimproved psychological problems may lead to extreme tendencies if they continue to grow. A portion of the research has addressed the impact of the COVID-19 pandemic on suicidal ideation and behavior among young people, but because it has not been adequately studied, this portion is shown in the blue dashed box. According to related studies, pediatric mental health-related ED visits among adolescents aged 12–17 years increased by 31% in the USA beginning in April 2020, compared with 2019 data (50). In May 2020, there was also an increase in the number of ED visits for suspected suicide attempts among young people aged 12–25 years (51). In Australia, an increase in the number of contacts regarding suicide/self-harm was also found through helplines used by children and adolescents (52). Of particular interest to us is the fact that an increase in suicide rates among young people was not found in some areas early in the outbreak (53, 54), suggesting that suicidal ideation and behavior among young people may also increase cumulatively over time during the epidemic (55, 56). Further research is still necessary to determine whether COVID-19 caused the rise in the suicide rate among young people.

###### Effect mechanism in cluster 4: Risk perception and practice toward the epidemic

It was found that young adults perceived a higher risk than older adults during the epidemic (57) and exhibited higher anxiety values (58). This higher risk perception may be related to the high exposure to COVID-19 information on social media and the emotions of anger and fear (59). Adolescents, on the other hand, showed a lower perception of risk, which could lead to their worse practice performance (60). Adolescence is a stage of life that experiences excitement and adventure, and as such, some adolescents may feel invulnerable and fail to comply with preventive measures. Also, there is a strong correlation between COVID-19 risk perception and vaccination status: individuals with a higher COVID-19 risk perception are more likely to be vaccinated. In addition, there are specific groups that differ in their risk perception. Some studies have shown that young people from disadvantaged socioeconomic groups still have a lower level of awareness and acceptance of COVID-19 (61, 62). Unfortunately, this gap is also reflected in studies of health disparities among young people facing COVID-19. Young people may differ in their vulnerability and susceptibility to COVID-19 depending on family conditions, race, etc. For example, children from low-income families (63) and black or Spanish children (64, 65) have significantly higher rates of COVID-19 infection and mortality. Research has found that youth who identify as sexual minorities experienced higher levels of disruption and adversity during the pandemic (66). And sexual minority youth are more vulnerable and affected than other cisgender and heterosexual peers (67, 68). There is a lack of research on such groups, which is shown in the green dashed box.

###### Effect mechanism in cluster 5: Interventions and social support

Social support could help young people face challenges. Positive teacher-student relationships can help young people in the student phase to effectively improve their mental health (69, 70). A survey found that more than two-thirds of teens said communication with teachers decreased during the pandemic (71). Enhancing students' school connectedness has been shown to have substantial protective effects on health and wellbeing during adolescence and adulthood (66). Therefore, those working in education should pay attention to this issue and increase student-teacher interaction to meet the social and emotional needs of students during COVID-19. Next, family relationships act as a double-edged sword that has a dual impact on young people who are closed to home. On the one hand, it is seen that many adolescents have experienced significant negative changes in their relationships with their parents, such as having more anger, arguments, and resentment, compared to the pre-pandemic period (72). And these poor parent-child relationships are risk factors for the development of anxiety and depressive symptoms (73). On the other hand, positive and stable family relationships help to alleviate mental health problems in young people (74). It was found that families with high levels of education were effective in alleviating adolescents' stress and emotional fluctuations during the COVID-19 pandemic, while adolescents from low/moderately educated families experienced more dramatic and negative changes in their emotional health (75). In addition, the emergence of telemedicine in a lockdown setting offers a new intervention to help young people respond to the COVID-19 pandemic and has proven to be effective (76, 77). Telemedicine and specific practices for dealing with various conditions deserve to be further explored in the future to support and help young people with adverse mental health conditions.

## Limitations

First, although this study tried to collect data as accurately and comprehensively as possible, the database selected for this paper was the Web of Science. There are also databases such as PubMed, Google Scholar, and Scopus that were not covered, which may lead to the omission of a small number of relevant articles. Second, this paper obtained the main research domains in this field by clustering keywords, which is not comprehensive. This is because it relies too much on keyword content analysis, but does not provide enough in-depth knowledge of specific reviews, which is the result of the shortcomings of bibliometrics. Therefore, future studies need to be combined to further elucidate how the COVID-19 pandemic has affected young people from multiple perspectives (including country, discipline, study design, etc.).

## Conclusion

Since the beginning of 2020, the number of publications exploring the relationship between COVID-19 and young people has increased, the scope of studies has expanded, and the content of studies has been refined. The current research in this area is mainly from North America and Europe, and systematic and comprehensive studies across regions still need to be explored. The effect of the COVID-19 pandemic on young people has been focused on mental health, as evidenced by a large body of literature. The research hotspots have gradually evolved from focusing on the superficial description in the early stages of the outbreak and the intuitive impact on young people to exploring more in-depth and detailed psychological problems and countermeasures.

In this study, the literature in this field was classified into five main research domains through keyword clustering analysis. Linking these five domains by four pathways helps us to reveal the mechanism of COVID-19's effect on young people and to identify research content that has not yet received much attention but has some promise for the future. The development of specific groups (young people from disadvantaged socioeconomic groups, sexual minority adolescents) and extreme tendencies (suicide) among young people during the COVID-19 period deserve further attention.

## Data availability statement

The original contributions presented in the study are included in the article/Supplementary material, further inquiries can be directed to the corresponding author.

## Author contributions

XL proposed the topic and designed the overall article. JY analyzed the data and wrote the article. All authors have read and agreed to the published version of the manuscript.

## Funding

This work was supported by the National Social Science Fund of China (Grant Number 22BGL223).

## Conflict of interest

The authors declare that the research was conducted in the absence of any commercial or financial relationships that could be construed as a potential conflict of interest.

## Publisher's note

All claims expressed in this article are solely those of the authors and do not necessarily represent those of their affiliated organizations, or those of the publisher, the editors and the reviewers. Any product that may be evaluated in this article, or claim that may be made by its manufacturer, is not guaranteed or endorsed by the publisher.
